# Primary pulmonary mucosa-associated lymphoid tissue lymphoma presenting as a right hilar mass: A case report

**DOI:** 10.1097/MD.0000000000051002

**Published:** 2026-10-02

**Authors:** Liyuan Fu, Taipeng Zeng, Chengkun Hong, Yuhang Zhang, Hao Huang

**Affiliations:** a Fuzong Teaching Hospital, Fujian University of Traditional Chinese Medicine (900th Hospital), Fuzhou, China; b College of Integrative Medicine, Fujian University of Traditional Chinese Medicine, Fuzhou, China.

**Keywords:** ^18^F-FDG PET/CT, case report, computed tomography imaging, maximum standardized uptake value, primary pulmonary lymphoma

## Abstract

**Rationale:**

Primary pulmonary lymphoma (PPL) is an extremely rare malignant lung tumor that is frequently misdiagnosed as pneumonia or lung cancer due to its atypical radiological and clinical presentation. The rationale of this report is to elucidate the diagnostic value of combining computed tomography (CT) and ^18^F-fluorodeoxyglucose positron emission tomography/computed tomography (^18^F-FDG PET/CT) in evaluating a rare presentation of PPL.

**Patient concerns:**

A 44-year-old male was admitted after an incidental lung lesion was discovered during routine screening. He was completely asymptomatic, lacking respiratory or systemic “B symptoms.”

**Diagnoses:**

A baseline chest CT identified a 3.9 × 3.5 cm soft-tissue mass in the right hilar region that compressed the right upper lobe bronchus, leading to secondary post-obstructive consolidation that lacked the characteristic air bronchogram sign. The CT also revealed an incidental 2.6 × 2.6 cm anterior mediastinal nodule. Subsequent whole-body ^18^F-FDG PET/CT demonstrated intense tracer uptake in the right hilar mass with a maximum standardized uptake value of 7.1 and mild-to-moderate uptake in an adjacent regional lymph node (maximum standardized uptake value of 3.2). The scan showed completely absent uptake in the anterior mediastinal nodule and the post-obstructive consolidation, arguing against metabolically active malignancy in those areas. Histopathological and immunohistochemical analysis from a bronchoscopic transbronchial lung biopsy confirmed primary pulmonary mucosa-associated lymphoid tissue lymphoma.

**Interventions:**

The patient underwent localized radiotherapy targeting the right hilar mass and the FDG-avid regional hilar/posterior mediastinal lymph node. A total dose of 66 Gy was delivered in 18 fractions over the course of 3 weeks.

**Outcomes:**

At the 2-month post-discharge follow-up, a repeat chest CT revealed a marked reduction in the size of both the hilar lesion and the regional lymph nodes. The patient remained completely asymptomatic, and his laboratory parameters normalized.

**Lessons:**

Differentiating PPL from central lung cancer and coexisting benign mediastinal abnormalities presents a significant clinical challenge, particularly when atypical radiological features mimic aggressive carcinomas. Whole-body ^18^F-FDG PET/CT serves as an invaluable, noninvasive modality for metabolic characterization, accurate loco-regional staging, and preventing clinical overstaging by effectively differentiating metabolically active lymphoma from concurrent benign lesions.

## 1. Introduction

Primary pulmonary lymphoma (PPL) is an uncommon subtype of extranodal lymphoma, originating from the lymphatic tissue within the lungs. It accounts for 3.6% of all extranodal lymphomas, 0.4% of all lymphomas,^[1]^ and represents 0.5% of all primary malignant tumors in the lungs.^[2]^ This disease originates from the bronchial mucosa-associated lymphoid tissue (MALT) or pulmonary lymphoid tissue. PPL can be classified into Hodgkin lymphoma and non-Hodgkin lymphoma, with Hodgkin lymphoma being less prevalent. Non-Hodgkin lymphoma can be further categorized into B-cell lymphomas, T-cell lymphomas, as well as some rare types such as plasma cell lymphoma and lymphomatoid granulomatosis. B-cell lymphoma represents the most prevalent form of PPL, with MALT being the predominant subtype, accounting for approximately 70% to 80% of all PPL cases.^[3,4]^ Next in line is diffuse large B-cell lymphoma, constituting 5% to 20% of all PPL cases.^[5]^

Pekelis provided the first account of peribronchial lymphosarcoma in 1931, marking the initial documentation of PPL in medical research.^[6]^ PPL is characterized by clonal abnormal proliferation of lung parenchyma or bronchial lymphoid tissue, with or without mediastinal lymph node enlargement, and absence of extrapulmonary lesions within 3 months from onset or diagnosis. Importantly, involvement of hilar or mediastinal lymph nodes is considered loco-regional (satellite) disease rather than true extrapulmonary spread. In the early stages, PPL typically presents with subtle clinical symptoms, and the timing of symptom onset remains uncertain. Furthermore, its clinical manifestations, such as cough, discomfort, fever, and fatigue, are characterized by atypical features and lack specific imaging findings.^[7]^ Due to the presence of atypical clinical symptoms and radiological features, accurate diagnosis of this disease poses a significant challenge and often leads to misdiagnosis as other pulmonary conditions such as pneumonia or lung cancer, thereby impacting clinical management. Here, we report a case of primary pulmonary MALT lymphoma presenting as a right hilar mass with secondary post-obstructive consolidation and a concurrent non-fluorodeoxyglucose (FDG)-avid anterior mediastinal nodule.

## 2. Case description

A 44-year-old male farmer was admitted to our hospital following the incidental discovery of a right upper lobe lesion during a routine annual health examination. In his region, annual low-dose chest CT is commonly performed as part of standard occupational and public health screening programs. Notably, his prior annual chest CT scans over the past few years had been consistently normal, confirming that the current lesion was a recent development. The patient was completely asymptomatic, denying any respiratory symptoms (e.g., cough, hemoptysis, chest pain) or systemic “B symptoms” (e.g., fever, night sweats, weight loss). His past medical, occupational, and family histories were unremarkable, with no history of smoking or exposure to chemical hazards.

Physical examination upon admission was entirely unremarkable, with clear bilateral breath sounds and no palpable superficial lymphadenopathy. Laboratory evaluations revealed mild thrombocytopenia (platelet count: 81 × 10^9^/L) and active hepatitis B virus infection (hepatitis B surface antigen positive, hepatitis B virus deoxyribonucleic acid: 1.15 × 10^7^ IU/mL). Other routine laboratory tests, as well as electrocardiography and echocardiography, showed no clinically significant abnormalities pertinent to his pulmonary presentation.

Baseline diagnostic chest CT demonstrated a 3.9 × 3.5 cm soft-tissue mass in the right hilar region (Fig. 1). The mass compressed and narrowed the right upper lobe bronchus, with associated distal post-obstructive consolidation/atelectatic change. A well-circumscribed anterior mediastinal nodule measuring 2.6 × 2.6 cm was also identified.

**Figure 1.**
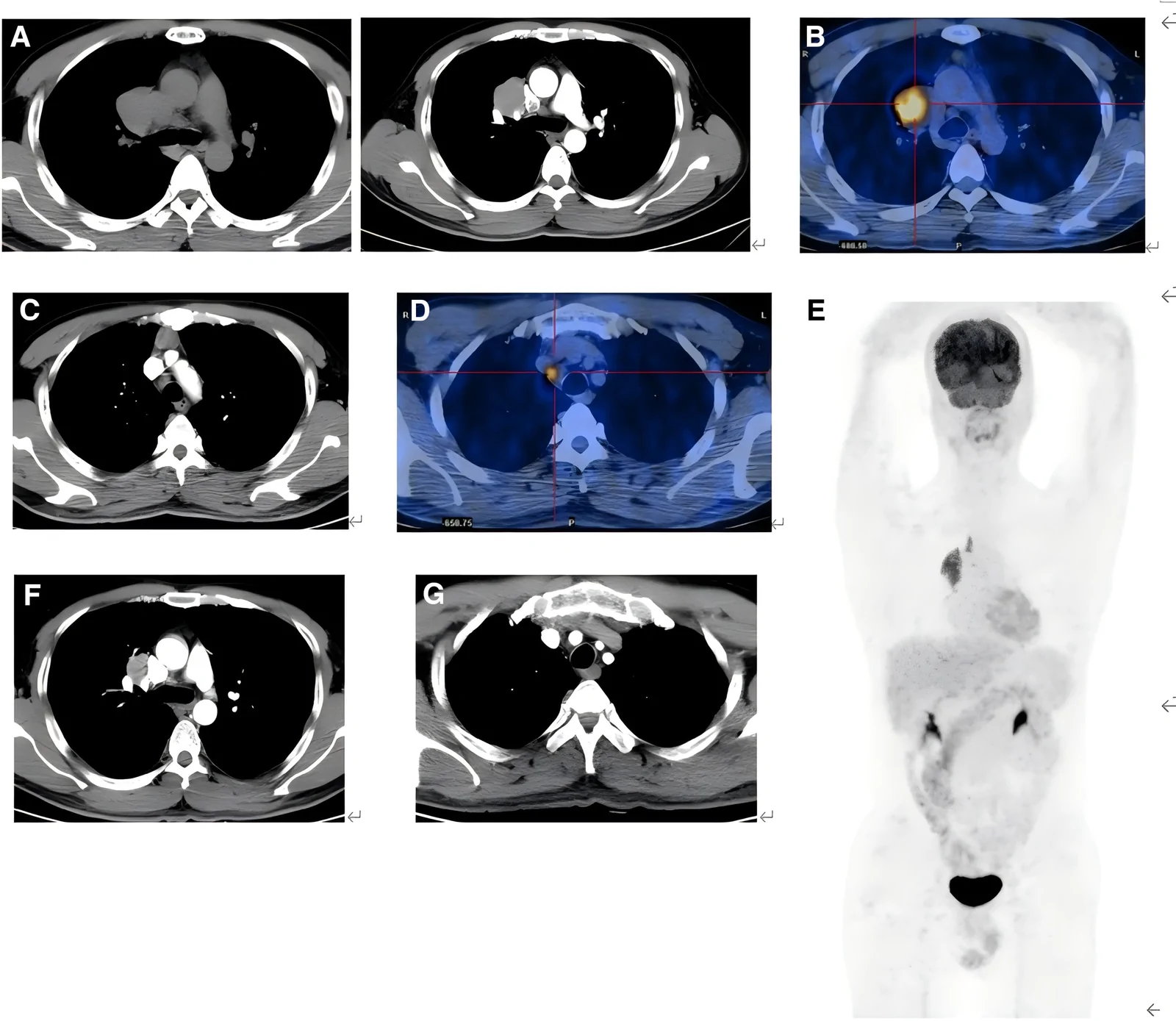
Imaging findings in a 44-year-old man with primary pulmonary MALT lymphoma. (A) Baseline diagnostic chest CT (mediastinal window). The unenhanced image (left) and contrast-enhanced image (right) show a 3.9 × 3.5 cm right hilar soft-tissue mass with mild homogeneous enhancement. The mass severely compresses and narrows the right upper lobe bronchus, leading to distal post-obstructive consolidation that notably lacks air bronchograms. (B) Axial fused PET-CT image shows increased FDG uptake in the right hilar mass, with an SUV_max_ of 7.1. (C) Baseline chest CT shows a regional hilar/posterior mediastinal lymph node measuring approximately 1.2 × 1.1 cm. (D) ^18^F-FDG PET/CT fusion image shows mild-to-moderate FDG uptake in this regional lymph node, with an SUV_max_ of 3.2. (E) Maximum-intensity-projection image shows FDG uptake in the right hilar mass and an adjacent mediastinal lymph node, without distant hypermetabolic lesions. (F) Follow-up chest CT 2 months after treatment shows interval reduction of the right hilar lesion to 2.3 × 1.7 cm. (G) Follow-up chest CT shows interval reduction of the regional lymph node to 0.7 × 0.6 cm. ^18^F-FDG = fluorine-18 fluorodeoxyglucose, CT = computed tomography, MALT = mucosa-associated lymphoid tissue, PET/CT = positron emission tomography/computed tomography, SUV_max_ = maximum standardized uptake value.

Whole-body ^18^F-FDG positron emission tomography/computed tomography (PET/CT) was subsequently performed for metabolic characterization and staging. The computed tomography (CT) component of the PET/CT was a low-dose, non-contrast CT used for attenuation correction and anatomical localization. PET/CT showed increased FDG uptake in the right hilar mass, with a maximum standardized uptake value (SUV_max_) of 7.1, and mild-to-moderate uptake in an adjacent regional hilar/posterior mediastinal lymph node, with an SUV_max_ of 3.2. Both SUV_max_ values were normalized to body weight. No distant hypermetabolic lesion was identified. The distal post-obstructive lung opacity and the anterior mediastinal nodule showed no abnormal FDG uptake, suggesting that these findings were unlikely to represent metabolically active lymphoma involvement.

Bronchoscopic transbronchial lung biopsy was subsequently performed to establish a pathological diagnosis. Histopathological examination showed dense infiltration of small atypical lymphoid cells. Immunohistochemistry demonstrated positivity for cluster of differentiation 20 and 79a and negativity for cytokeratin and thyroid transcription factor 1, supporting the diagnosis of extranodal marginal zone lymphoma of MALT, also known as pulmonary MALT lymphoma.

After the diagnosis was confirmed, localized radiotherapy was initiated. Table 1 summarizes the clinical timeline from lesion detection to diagnosis and treatment initiation.

**Table 1:** Timeline of events from lesion detection to diagnosis and treatment.

Date/period	Event
10 d before admission	Initial non-contrast chest CT at a local hospital revealed a right upper lobe space-occupying lesion during routine annual screening.
5 d before admission	Outpatient diagnostic chest CT at our hospital suggested a right hilar mass with secondary obstructive changes; patient admitted for further evaluation.
July 5, 2017	Patient formally admitted to the hospital.
July 7, 2017	Whole-body ^18^F-FDG PET/CT (low-dose CT for attenuation correction) performed: showed high uptake in the right hilar mass (SUV_max_: 7.1) and regional lymph nodes, with no distant hypermetabolic disease.
July 7, 2017	Bone marrow biopsy performed; ruled out systemic lymphoma infiltration.
July 17, 2017	Following lung biopsy confirmation of MALT lymphoma, localized radiotherapy was initiated (66 Gy in 18 fractions over 3 wk).
During radiotherapy	Hematopoietic support provided for preexisting thrombocytopenia; antiviral therapy managed HBV infection.
2 mo post-discharge	Follow-up visit: patient asymptomatic. Platelet count normalized, HBV DNA significantly reduced. Repeat chest CT showed a marked reduction in the size of the hilar lymphoma lesion.

No extrapulmonary lymphoma involvement was identified at diagnosis or during the subsequent 3-month follow-up period.

18 F-FDG = fluorine-18 fluorodeoxyglucose, CT = computed tomography, DNA = deoxyribonucleic acid, Gy = gray, HBV = hepatitis B virus, MALT = mucosa-associated lymphoid tissue, PET/CT = positron emission tomography/computed tomography, SUV_max_ = maximum standardized uptake value.

## 3. Discussion

Malignant lymphoma encompasses a spectrum of malignancies originating from lymph nodes and other lymphatic tissues, with a higher incidence of secondary involvement in the lungs; however, PPL is relatively infrequent. PPL constitutes merely 3% to 4% of all extranodal lymphomas and <0.5% of primary malignant lung tumors.^[4]^ Currently, the definition of PPL encompasses a clonal lymphoproliferative disorder primarily affecting one or both lungs, including the parenchyma and bronchi, while excluding invasion into extrapulmonary tissues at the time of diagnosis and within 3 months thereafter.^[4,8]^ PPL frequently presents with atypical respiratory symptoms, including cough, sputum production, hemoptysis, chest pain, and dyspnea. In addition, systemic manifestations such as fever, night sweats, and weight loss may also be observed.^[9]^ However, these clinical manifestations do not significantly contribute to its diagnosis. Previous studies have demonstrated that approximately 37% of patients are diagnosed without experiencing any prodromal symptoms.^[10]^ A feature consistent with the present patient was a lung space-occupying lesion during a routine physical examination, with no respiratory or systemic symptoms at initial presentation. Multislice spiral CT is currently the preferred evaluation method for PPL due to its enhanced ability to provide precise and comprehensive visualization of lesion morphology and density, thereby facilitating radiologists in identifying and differentiating even smaller lesions.^[11]^ The radiological manifestations of PPL are intricately linked to its underlying pathology. When tumor cells infiltrate the alveoli, they can manifest as adenoid-like nodules. Diffuse infiltration or invasion of interlobar fissures can lead to extensive consolidation resembling pneumonia. Gradual extensive infiltration of tumor cells into the alveolar spaces may result in the formation of nodules, masses, or consolidations of varying sizes. Interstitial changes may occur when tumor cells diffusely infiltrate the interlobar septa, pulmonary lobules, and alveolar septa; involvement of the surrounding interstitium can cause bronchial deformation and dilation. Further enlargement of the tumor can induce ischemia and necrosis at its core, leading to cavity formation. Thickening of visceral and parietal pleura or the presence of pleural effusion may be observed upon invasion by tumor cells.^[11–13]^ Notably, the present patient’s PPL (MALT lymphoma subtype) exhibited a distinct mass-like CT pattern: a 3.9 × 3.5 cm right hilar soft-tissue mass with homogeneous density (CT value: 41 HU) and clear, smooth margins – consistent with the “nodular or mass-like type” of PPL, but with a unique association: an anterior-superior mediastinal nodule (2.6 × 2.6 cm, CT value: 66–72 HU) suspected to be a thymoma. This concurrent mediastinal lesion is rare in PPL cases, as PPL typically involves only the pulmonary parenchyma/bronchi without extrapulmonary associations at diagnosis, making the patient’s presentation unusual.

Previous studies have consistently demonstrated that the distribution of PPL does not exhibit any predilection for specific lung lobes and can manifest as a solitary or multiple lesion. In rare instances, it may extend across different pulmonary lobes.^[14]^

Reported CT manifestations of PPL are heterogeneous and may include nodules or masses, consolidation, interstitial infiltration, or mixed patterns.^[15]^ In the present case, the lesion appeared as a right hilar soft-tissue mass with secondary post-obstructive consolidation, a pattern that closely mimicked central bronchogenic carcinoma. The absence of a typical air bronchogram, together with marked narrowing of the right upper lobe bronchus, further increased the diagnostic difficulty. These findings suggest that CT morphology alone may be insufficient for differentiating PPL from lung cancer and that integrated assessment using metabolic imaging and histopathological confirmation is essential.

^18^F-FDG PET/CT is a well-established, guideline-recommended imaging modality. In comparison with conventional imaging techniques, it provides more precise diagnosis, staging, and treatment response assessment for tumors.^[16,17]^ This modality not only yields detailed anatomical information but also evaluates the tumor’s biological characteristics at a metabolic level.^[18]^

The diverse imaging features exhibited by PPL pose a challenge in distinguishing it from other pulmonary diseases, including lung cancer, tuberculosis, and lobar pneumonia. It is crucial to note that relying strictly on numerical SUV_max_ cutoffs to differentiate PPL from primary lung cancer can be highly misleading, as significant metabolic overlap exists depending on the specific histologic subtypes and proliferative indices.^[19,20]^ Instead of strict thresholding, the present patient’s ^18^F-FDG PET/CT revealed uniquely characteristic metabolic patterns: the right hilar MALT lymphoma mass exhibited intense yet relatively homogeneous radiotracer uptake (SUV_max_: 7.1) without signs of massive central necrosis, a morphological feature more frequently observed in aggressive lung cancers. Furthermore, pneumonia typically presents with acute infectious symptoms (e.g., fever, leukocytosis) and diffuse inflammatory uptake, whereas our patient was completely asymptomatic with no abnormal FDG uptake in the post-obstructive distal lung opacities.

Finally, PET/CT played a critical role in accurately staging this patient by confirming that the concurrent anterior mediastinal nodule showed virtually no abnormal FDG uptake. This crucial metabolic differentiation effectively ruled out regional malignant spread, confirming its benign nature (compatible with a suspected thymoma). This finding successfully prevented clinical overstaging and facilitated the highly effective, localized radiotherapy plan.

## 4. Conclusion

PPL has heterogeneous radiological manifestations and nonspecific clinical symptoms, which may lead to misdiagnosis as lung cancer, pneumonia, or tuberculosis, especially when classic imaging features such as the air bronchogram are absent. In the present case, ^18^F-FDG PET/CT provided useful metabolic information for lesion characterization, baseline staging, and treatment planning by identifying FDG-avid lymphoma involvement in the right hilar mass and regional lymph node while showing no distant hypermetabolic disease. Integrated interpretation of CT morphology, PET metabolic findings, and histopathological confirmation remains essential for accurate diagnosis and appropriate management of PPL.

## Author contributions

**Conceptualization:** Taipeng Zeng.

**Formal analysis:** Liyuan Fu, Taipeng Zeng.

**Funding acquisition:** Liyuan Fu, Hao Huang.

**Investigation:** Liyuan Fu, Hao Huang.

**Methodology:** Liyuan Fu.

**Project administration:** Liyuan Fu, Taipeng Zeng.

**Resources:** Liyuan Fu, Yuhang Zhang.

**Supervision:** Chengkun Hong.

**Validation:** Chengkun Hong.

**Visualization:** Chengkun Hong.

**Software:** Yuhang Zhang.

**Writing – original draft:** Taipeng Zeng.
